# The viral set point in primary HIV infection is associated with specific amino acids in position 97 of MHC class I

**DOI:** 10.1186/1742-4690-9-S2-P163

**Published:** 2012-09-13

**Authors:** SA Vaidya, H Streeck, F Pereyra, ES Rosenberg, BD Walker, M Altfeld

**Affiliations:** 1 Ragon Institute of MGH, MIT, and Harvard, Boston, MA, USA

## Background

Sequence variations which affect the peptide binding groove of the major histocompatibility complex (MHC) class I allele HLA-B are strongly associated with viral control in chronic HIV infection. We sought to determine if these differences affect primary HIV infection and establishment of the early viral set point.

## Methods

We longitudinally followed 428 individuals during primary HIV infection. In 110 individuals, we were able to determine the viral set point at six months following diagnosis. Associations between known genetic polymorphisms in HLA class I residues and HIV viral load and viral set point were identified.

## Results

The identity of the amino acid at position 97 in the peptide binding groove of HLA-B was significantly associated with the level of initial viremia in acute infection (p=0.04) and the viral set point (p=0.025). Individuals with valine at position 97 had a nearly 10-fold lower mean viral set point than those with serine. This association was dependent on presence of the B*57 allele, which was also associated with a lower viral set point. The initial viral load at the time of primary HIV diagnosis was closely correlated with the level of the subsequent viral set point (R=0.4, p=<0.0001).

## Conclusion

Control of HIV during acute infection and the viral set point are strongly associated with the amino acid in position 97 of HLA-B. These results provide evidence for genetic predictors of HIV control in primary infection and highlight the importance of sequence polymorphisms in the binding pocket of MHC class I which may be relevant to the development of future HIV vaccines.

